# Underemployment, Work Needs, and Job Satisfaction: Does Social Support Matter?

**DOI:** 10.3390/bs14040335

**Published:** 2024-04-17

**Authors:** Furkan Kirazci, Aysenur Buyukgoze-Kavas

**Affiliations:** Department of Educational Sciences, Faculty of Education, Ondokuz Mayis University, Samsun 55139, Türkiye; aysenur@omu.edu.tr

**Keywords:** underemployment, Turkish employees, psychology of working theory

## Abstract

Global problems that have emerged in recent years have caused an increase in underemployment rates, especially in developing countries. Researchers emphasize that underemployment has as many negative consequences as unemployment on well-being. In order to examine the variables that may buffer these consequences, we draw on the Psychology of Working Theory to propose a model in which a mediating role of psychological needs and a moderating role of social support are assumed in the relationship between underemployment and job satisfaction. We collected and analyzed data from 459 Turkish employees (181 women and 278 men) and found that underemployment was negatively related to job satisfaction and that work needs satisfaction mediated the relationship between underemployment and job satisfaction. Further, social support moderated the relationship between subjective underemployment and job satisfaction, so it was insignificant when social support was higher. These findings provide researchers and practitioners with a different perspective on underemployment.

## 1. Introduction

Across the globe, wars, economic recessions, refugee flows, and pandemics have had dramatically devastating effects on the world of work [1,2]. Notably, the COVID-19 pandemic weakened the economies of many countries regardless of their development status [3]. On the one hand, it resulted in job loss; on the other, it increased the rate of precarious work (such as short-term, unstable, and insecure work) [4,5]. More specifically, this negative atmosphere in the global economy has caused an increase in underemployment rates, especially in developing countries [2,6,7], with researchers arguing that underemployment will remain a problem in the global economy after the pandemic [8]. However, because psychology scholars tend to focus on unemployment rather than underemployment, the latter remains understudied [9]. This is a particular concern because underemployment has at least as many negative consequences as unemployment on well-being [9,10,11] and work fulfillment [12,13,14]. 

In addition to traditional economic factors, the advent of artificial intelligence (AI) technologies has introduced new complexities to the employment landscape. AI has the potential to increase underemployment rates by automating routine tasks previously performed by humans, leading to displacement or deskilling of workers [15]. This phenomenon, often referred to as “technological unemployment”, can exacerbate underemployment, particularly among low-skilled or routine-based jobs [16]. For instance, a study by Frey and Osborne estimated that up to 47% of total US employment is at risk of being automated in the coming decades, potentially leading to a significant rise in underemployment [17].

**Figure 1:**
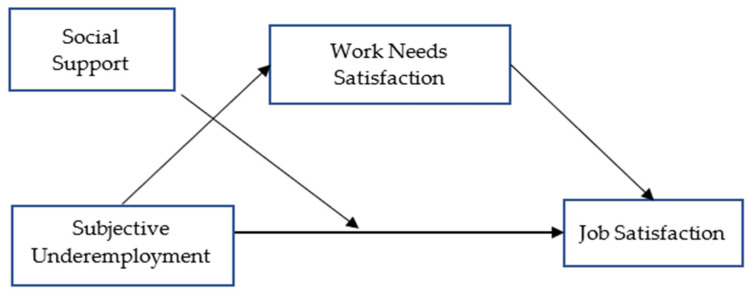
Hypothesized model.

In recent years, underemployment has been examined through different models under different approaches, such as person–environment fit or latent deprivation theory [12,18]. For example, Kim and Allan explored how psychological needs mediate between underemployment and the perception of meaningful work [13]. The results showed that the indirect effect of underemployment on meaningful work via autonomy (a subdimension of psychological needs) was significant. Another study by Allan and colleagues examined whether meaningful work had a significant moderating effect on the relationship between underemployment and well-being [12]. Contrary to their expectations, the results showed that meaningful work did not buffer the negative effect of underemployment on well-being. Rather, the researchers found that meaningful work was associated with a positive relationship between underemployment and lower well-being. Consequently, researchers have emphasized the importance of the variables that may be effective in mediating or buffering these negative consequences of underemployment [12]. Therefore, based on the Psychology of Working Theory [19], we introduce a model wherein the mediating function of work needs and the moderating influence of social support are posited in the correlation between underemployment and job satisfaction (Figure 1).

### 1.1. Underemployment and Türkiye

Underemployment, a multifaceted construct, has been defined and operationalized in diverse manners by researchers [20,21]. It is generally defined as a type of employment that is inferior to full employment in terms of selected standards [21,22]. Additionally, underemployment is considered a multi-dimensional construct comprising various components. Scholars have identified seven dimensions of underemployment: “overqualification (possessing more education or skills than a job requires)”, “field mismatch (being involuntarily employed outside of one’s area of education or experience)”, “involuntary part-time work (involuntarily working in a part-time job)”, “involuntary temporary work (involuntarily working in a temporary position)”, “status underemployment (having a lower status than in one’s previous job or than people with similar qualifications)”, “underpayment (earning less income than in one’s previous job or than people with similar qualifications)”, and “poverty-wage employment (earning insufficient income to meet one’s basic needs)” [20,22]. Allan and colleagues developed the Subjective Underemployment Scale using these dimensions based on subjective perception and found that these dimensions correlated with each other and loaded onto a general factor [20]. Consequently, we will use this approach to underemployment in the present study.

As mentioned above, underemployment is a common problem across the globe and is projected to continue to be an issue in the future [2,8]. Global issues, especially in recent decades, have caused rising underemployment rates, particularly in developing countries such as Türkiye [2]. Due to its geopolitical position, the Republic of Türkiye is both European and Asian. The country’s population is estimated to be around 85 million people, with young people constituting 15.1 percent of the total population [23]. According to the United Nations’ classification, Türkiye is a developing economy and is among the upper-middle-income countries in terms of gross national income (GNI) per capita [24]. It is an economically promising country owing to its young population and vision for the future. However, in recent years, Türkiye has faced many challenges that have devastated its economy, including an influx of refugees, terrorism, its currency’s dramatic depreciation, the COVID-19 pandemic, and the war between Russia and Ukraine [25,26,27]. In this context, statistical data show that Türkiye’s economy is in a precarious situation. For example, the increase in the CPI (consumer price index) in December of 2023 was 64.77%; in January of 2024, it was 64.86%, and, on the 12 months moving average basis, it was 54.72% in January 2024. Moreover, the CPI annual rates of change in four critical groups, food and non-alcoholic beverages (69.71%), transportation (77.54%), health (78.57%), and education (79.81%), were above average [28]. To deal with high inflation, the Turkish government raised the minimum wage by 49% in January 2024. Effective from January 2024, the new minimum wage was set as TRY 17.002 (about USD 554) monthly. However, inflation continuing to rise at the current pace, just like in previous years, will cause the rate of wage increases to fall below the annual CPI increase within a few months. The rapid increase in inflation has led to the devaluation of many workers’ current earnings. Consequently, many workers have experienced underemployment because of high inflation. Moreover, labor force statistics reports revealed that underutilization rates (time-related underemployment) increased from 21.9% to 24.7% in 2023 [29]. Further, irregular increases in the number of university graduates and supply–demand imbalance in the highly educated workforce are other factors contributing to the increasing underemployment rates in Türkiye [30,31]. In summary, although underemployment cannot be measured directly, employment trends in Türkiye signify that underemployment has increased dramatically.

### 1.2. Theoretical Background

In explaining underemployment, researchers have benefited from various theories, such as “Latent Deprivation Theory”, “Human Capital Theory”, “Relative Deprivation Theory”, and “Person–Job Fit Theory” [12,21]. These theories mainly clarify the causes and consequences of underemployment [20]. However, recent research has focused on underemployment mediated and moderated by various variables and has attempted to test underemployment under various models [12,13,18]. Similar to the research conducted by Kim and Allan [13], we propose that underemployment can be addressed based on the Psychology of Working Theory (PWT), which aims to explain the predictors and outcomes of decent work from a multicultural perspective in disadvantaged groups and across different cultures [19]. At the core of the PWT is “decent work”, a multidimensional concept that has five dimensions: (a) “physically and mentally safe working conditions”, (b) “sufficient compensation”, (c) “access to health care”, (d) “hours that allow adequate rest and free time”, and (e) “organizational values that integrate family and social values” [19,32]. According to the PWT, securing decent work meets work needs and results in job satisfaction [19]. Decent work and underemployment are not synonymous, but underemployment may relate to job satisfaction with the mediated role of work needs satisfaction, similar to decent work, as decent work signifies the fundamental benchmark for employment. In contrast, underemployment denotes work falling short of a reasonable standard [13]. In other words, if we imagine employment on a continuum, with decent work at one end and unemployment at the other, underemployment seems to be close to the unemployment side.

### 1.3. Subjective Underemployment and Job Satisfaction

Job satisfaction is described as the pleasant emotional response derived from evaluating one’s job, reacting emotionally to it, and forming an attitude toward it [33]. Moreover, extensive research in the literature demonstrates numerous positive outcomes associated with job satisfaction for organizations and employees. Consequently, while job satisfaction contributes to employee happiness, life satisfaction, and positive affect [34], it also fosters heightened organizational performance and facilitates smoother alignment with organizational objectives [35]. However, job satisfaction may decline due to job–role incompatibility or unmet job-related expectations. Research indicates that experiences of underemployment among employees have a detrimental effect on their job satisfaction [21]. Consequently, studies have demonstrated that perceptions of time-related underemployment, such as involuntary part-time work [36,37] and underutilization [38], adversely influence job satisfaction. Despite numerous studies investigating the relationship between underemployment and job satisfaction, the effective mechanisms contributing to the negative impact of underemployment on job satisfaction still need to be clarified. Consequently, as outlined in the introduction, this study seeks to explore the mediating and moderating variables influencing the impact of underemployment on job satisfaction.

### 1.4. Work Needs Satisfaction as a Mediator

According to the PWT, work should meet three basic needs: “the need for survival”, “the need for social contribution”, and “the need for self-determination” [19,39,40]. The need for survival refers to basic needs, such as food, shelter, and social capital, that guarantee life [19]. Social contribution refers to the ways in which individuals contribute to the welfare of societies through their work and connect with the broader social world [41]. Finally, the need for self-determination has been defined as the experience of engaging in intrinsically or extrinsically motivated activities in a meaningful and self-regulating way [19]. Self Determination Theory (SDT) considers a broad range of self-determination needs, including “autonomy”, “competence”, and “relatedness” [42]. Autonomy is “the need for individuals to act of their own will and feel free” [42]. Competence is “the need to improve one’s skills and sense of mastery in a relevant area” [41]. Finally, relatedness refers to “the need to feel a sense of belonging to a group” [43]. According to SDT, individuals feel internally motivated if they fulfill all three needs, enjoying personal growth, integrity, and well-being [43]. According to the SDT, meeting three basic needs (autonomy, competence, and relatedness) is essential for psychological health and well-being [42]. SDT-based research reveals that meeting people’s needs improves overall well-being [34]. Similarly, various studies have found a positive association between work needs and job satisfaction [44,45,46,47]. The satisfaction of work needs, which has been explored based on the Psychology of Working Theory, is associated with various positive variables, such as life satisfaction, decent work, and job satisfaction [41,48]. Therefore, we assume that work needs satisfaction will positively predict job satisfaction, as is indicated by previous research. The Psychology of Working Theory posits that satisfaction of work needs arises from attaining decent work [19]. Similarly, according to the SDT, non-optimal working conditions can inhibit individuals’ basic and psychological needs [49]. In the models proposed by Kalleberg, job rewards obtained by individuals from their employment, including financial, interpersonal, and intrinsic rewards, play a pivotal role in determining their job satisfaction by indicating fulfilling work-related needs [50]. In short, work needs satisfaction is closely related to the quality of employment. Therefore, we believe that there will be a negative correlation between underemployment and work needs satisfaction because underemployment, which is positioned between no employment and full employment, can also be characterized as a type of poor-quality employment [51]. Consequently, we assume that underemployment experience will inhibit employees’ work needs satisfaction, negatively affecting their job satisfaction.

### 1.5. Social Support as a Moderator

Social support (support from family, friends, or people who have an essential place in one’s life) is considered a resource that can be used in stressful situations [52] and which has a suppressing effect on stressful situations, increases one’s ability to cope, and reduces the severity of responses to stressful situations [53,54]. In the PWT, social support was also positioned as a moderator variable that could help effectively cope with stress and challenges associated with marginalization and economic constraints [19]. Creed and Moore examined the stress levels of underemployed people and investigated the role of social support on stress [55]. They revealed that underemployed people experienced less stress as they claimed to have higher social support. Further, talking about one’s thoughts and feelings about stress can increase one’s integration with respect to stressful experiences. In other words, talking about stress factors and receiving environmental support can help one develop a more optimistic outlook on stress factors [56,57]. We assume that underemployment experience is a stress factor that has a negative effect on job satisfaction. However, social support can buffer the negative effect of underemployment on job satisfaction. Studies have indicated that the social support employees experience from their environment and colleagues improves their work satisfaction [58,59]. Moreover, the results of Dooley and colleagues’ longitudinal study indicate that social support received from spouses softens the relationship between underemployment and depression and emphasizes the important role of social support in stressful situations such as underemployment and similar stressful situations [51]. Consequently, in the current study, we positioned social support as a moderator variable that can buffer the negative impact of underemployment on job satisfaction.

### 1.6. Present Study

The primary objective of the present study was to investigate how work needs satisfaction mediates the relationship between subjective underemployment experiences and job satisfaction. Testing this mediation effect will help us understand how employees’ underemployment experiences affect their job satisfaction. Our second aim in this research was to explore the moderating effect of social support on the correlation between underemployment and job satisfaction. Therefore, we aimed to determine whether social support played a buffering role regarding the negative effect of underemployment on job satisfaction. To this end, we tested two hypotheses in our current study:

**Hypothesis** **1.** *Subjective underemployment negatively relates to job satisfaction via work needs satisfaction.*

**Hypothesis** **2.** *Social support moderates the relationship between subjective underemployment and job satisfaction such that the relationship is not significant when social support is higher.*

## 2. Materials and Methods

After obtaining approval from the Institutional Ethics Board, data were collected from 466 working adults through both Google forms and paper-and-pen surveys. We distributed our survey link through various social media groups, including WhatsApp groups of diverse employees and LinkedIn connections. This approach allowed us to collect data from employees working in different organizations and workplaces. To ensure data integrity and prevent multiple submissions from the same participant, we activated the email feature in Google Forms, which limited responses to one unique email address. To gather data via paper-and-pen surveys, the researchers distributed scale forms to both public institutions and private businesses, subsequently collecting data directly at the workplaces. Finally, 239 data were acquired through online surveys, while an additional 227 were obtained via paper-and-pen surveys. When comparing the data gathered through online surveys with those obtained via paper and pen, it was determined that there existed a comparable distribution concerning demographic variables. All participants were required to complete a consent form, and they were informed that they had the option to withdraw from the study at any point without facing any penalties. Additionally, we did not offer any incentives or compensation to participants, and we established two criteria for participant selection. Firstly, eligible employees had to be at least 18 years old, and, secondly, they had to possess a minimum of two years of work experience. To gauge participants’ attention and bolster data reliability, we incorporated two distinct control questions alongside the survey queries (e.g., “Please mark two when you read this item”). Seven participants only responded to questions concerning demographic items and were thus removed, which left a final data size of 459.

The study involved 459 Turkish working adults with an average age of 36.03 years (*SD* = 9.38, range = 18–73). Participants self-identified as female (39.4%, n = 181) and male (60.6%, n = 278). A wide range of occupations were represented in the sample, including 71 different job titles. The most frequently reported job titles were teacher (26.4%, n = 121), sales advisor (6.5%, n = 30), clerk (4.6%, n = 21), worker (manual laborer, such as an electrician or mechanic) (3.7%, n = 17), engineer (2.8%, n = 13), nurse (2.8%, n = 13), academic staff (professor (n = 4), associate professor (n = 3), or research assistant (n = 3)) (2.2%, n = 10), and cashier (2.0%, n = 9). The participants’ job tenure varied from 2 to 50 years, with an average of 13.70 years (*SD* = 8.08, range = 2–50). Additionally, the study participants exhibited a diverse range of educational backgrounds. The educational distribution was as follows: 20 participants (4.4%) had completed elementary school, 80 participants (17.4%) had attained a high school education, 71 participants (15.5%) had received vocational training, 236 participants (51.4%) held a college degree, 45 participants (9.8%) had achieved a master’s degree, and 7 participants (1.5%) had earned a doctoral degree.

### 2.1. Instruments

#### 2.1.1. Underemployment 

We used the 37-item Subjective Underemployment Scale [20] to measure the underemployment perceptions of working adults. The SUS consists of six dimensions, including “pay”, “status”, “field”, “involuntary part-time work”, “involuntary temporary work”, and “poverty-wage employment.” The items of the scale are answered according to a scoring system ranging from 1 (strongly disagree) to 7 (strongly agree). Example items include “The income from my job is not enough” and “I deserve a higher position in my company”. Allan and colleagues reported that the SUS was positively correlated with withdrawal intentions and overqualification and negatively correlated with job satisfaction, meaningful work, and career commitment [17]. In addition, researchers found that subdimensions of the scale scores ranged from 0.95 to 0.97. The Turkish version of the SUS indicated good internal consistency between 0.87 and 0.96 [60]. In the current study, the internal consistency reliabilities for subdimensions of the scale ranged from 0.85 to 0.96.

#### 2.1.2. Work Needs Satisfaction

We assessed work needs satisfaction using the 20-item Work Needs Satisfaction Scale (WNSS), which was developed by Autin and colleagues in 2019. The WNSS consists of five subdimensions measuring “survival needs”, “social contribution needs”, “competence needs”, “related needs”, and “autonomy needs.” Participants answer the items of the scale on a 7-point Likert scale ranging from 1 (strongly disagree) to 7 (strongly agree). Each item begins with the same phrase “My work allows me to…”, and example items include “Have the resources to provide nutritious food for myself and my family” and “Feel like I am good at my job”. Autin and colleagues reported that the WNSS was positively correlated with decent work, job satisfaction, and life satisfaction [41]. In addition, researchers found that subdimensions of the WNSS’s internal consistency ranged from 0.85 to 0.95. Similar to the original scale development study [41], Kim and colleagues reported that the Turkish version of the scale’s estimated internal consistency was between 0.95 and 0.98 [61]. In the present study, the internal consistency of the scale’s total score was calculated at 0.92, and factor scores ranged from 0.80 to 0.91.

#### 2.1.3. Job Satisfaction 

To measure working adults’ satisfaction with their current jobs, we used the Job Satisfaction Scale developed by Judge and colleagues [62]. The Job Satisfaction Scale comprises five items, with responses recorded on a seven-point Likert scale, ranging from 1 (strongly disagree) to 7 (strongly agree). Sample items include “I find real enjoyment in my work” and “I feel fairly well-satisfied with my present job”. The estimated internal consistency of the scale was calculated at 0.88 [62]. Many studies found that the Job Satisfaction Scale has high internal consistency [25,63]. The Turkish version of the scale’s estimated internal consistency was also reported as 0.78 [64]. In the present study, the scale’s internal consistency was calculated at 0.81.

#### 2.1.4. Social Support 

To gauge the social support perceptions of working adults, we employed the “Multidimensional Scale of Perceived Social Support” (MSPSS) developed by Zimet and colleagues [65]. The 12-item MSPSS indicates working adults’ perceptions of social support from their friends, family, and significant others. Respondents provide answers to the scale’s 12 items using a seven-point Likert scale, ranging from 1 (strongly disagree) to 7 (strongly agree). Subdimensions of the scale have been named as family (example item: “I get the emotional help and support I need from my family”), friends (example item: “I have friends with whom I can share my joys and sorrows”), and a special person (example item: “There is a special person who is around when I am in need”). Zimet and colleagues found that subdimensions of the scale’s internal consistency ranged from 0.85 to 0.91 [65]. Eker and colleagues found similar results for the Turkish version of the MSPSS, with the estimated internal consistency ranging from 0.80 to 0.95 [66]. In the current study, the internal consistency of the scale’s total score was computed at 0.90.

## 3. Results

Before proceeding to formal model testing, preliminary analyses were conducted to ensure the quality and reliability of the data. First, we assessed the data for outliers using Tabachnick and Fidell’s guidelines [67], and none approached the Mahalonobis distance of the variables greater than the critical chi-square values (*x*^2^ = 16.266). Second, we assessed skewness and kurtosis to test the normality assumption and found that both skewness and kurtosis were within the acceptable range (Table 1) for all variables according to Weston and Gore [68]. Additionally, we visually inspected the histograms and found them to be normally distributed.

Additionally, we assessed multicollinearity and singularity—another assumption of the multiple regression. Multicollinearity and singularity occur when variables are too highly correlated (*r* = 0.90 and above; [67]). As can be seen in Table 1, there are moderate (*r* = 0.43, *r* = 0.41) and high (*r* = 0.50) correlations between dependent and independent variables. We conducted checks for multicollinearity utilizing tolerance and the variance inflation factor (VIF). All tolerance values were below 0.10, and all VIF values approached 1, indicating that multicollinearity was not a concern in this study [69,70].

**Figure 2:**
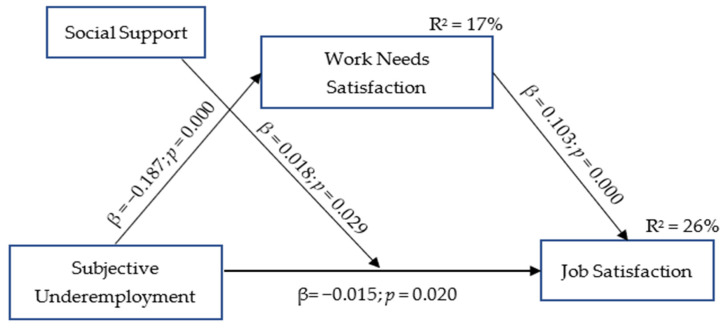
Hypothesized model with actual results.

After conducting preliminary analyses, we tested our hypotheses through a series of additional analyses. First, to test the mediation effect of work needs satisfaction’s five factors (survival needs, social contribution, competency, relatedness, and autonomy), we tested a structural equation model using AMOS 24. According to the literature, a good model–data fit is indicated by an *x*^2^/df value below 3; a Root Mean Square Error of Approximation (RMSEA) value below 0.08; and Comparative Fit Index (CFI), Goodness of Fit Index (GIF), and Tucker–Lewis Index (TLI) values close to 0.95 [68,71]. However, our model did not have a good fit to the data: *x*^2^/df = 42.464, RMSEA = 0.300, CFI = 0.280, GFI = 0.594, TLI = 0.512. Second, we used the work needs satisfaction scale total score as a mediator in our model, and it had a good fit to the data: *x*^2^/df = 1.344, RMSEA = 0.027, CFI = 0.998, GFI = 0.998, TLI = 0.995. Finally, to test our mediation and moderation hypotheses, we calculated the mean of the social support variable, converting it into a binary categorical variable, namely, low or high social support. We then utilized model 5 of the PROCESS macro developed by Hayes in SPSS 22 [72]. The results of our analyses are presented in Table 2 and Figure 2. 

As shown in Table 2, this model explained 26% of the variance in job satisfaction (R^2^ = 0.26, F(5-459) = 26,609, *p* < 0.001). Moreover, subjective underemployment negatively predicted job satisfaction (*β* = −0.015, SE = 0.007, 95% CI = [−0.028, −0.002]), and work needs satisfaction positively predicted job satisfaction (*β* = 0.103, SE = 0.010, 95% CI = [0.083, 0.124]). Additionally, we included job tenure and gender as covariate variables in the analysis. We concluded that neither job tenure (*β* = 0.005, SE = 0.026, 95% CI = [−0.046, 0.057]) nor gender (*β* = −0.609, SE = 0.430, 95% CI = [−1.454, 0.236]) made a significant contribution to the model.

We proposed that work needs satisfaction (Hypothesis 1) mediates the relationship between subjective underemployment and job satisfaction. The results of our research also revealed a mediation of work needs satisfaction (*β* = −0.019, SE = 0.029, 95% CI = [−0.026, −0.014]) between subjective underemployment and job satisfaction, supporting Hypothesis 1, because zero was not included in the confidence interval. Furthermore, the regression results of the model indicated that subjective underemployment was significant in predicting job satisfaction, supporting Hypothesis 2, that social support moderated the relationship between subjective underemployment and job satisfaction.

**Figure 3:**
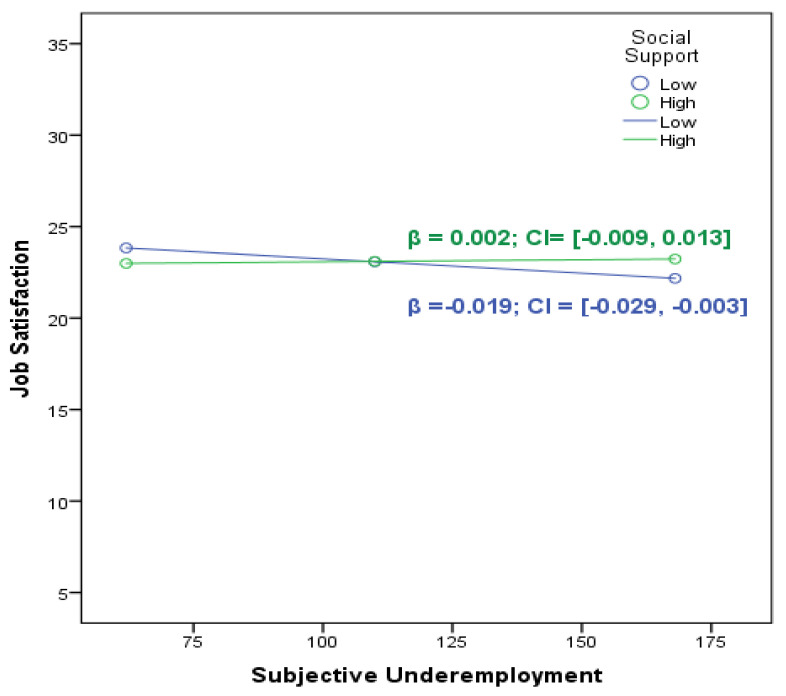
Social support as a moderator between subjective underemployment and job satisfaction.

As depicted in Table 2 and Figure 3, social support moderated the association between subjective underemployment and job satisfaction. Consequently, the previously negative and significant correlation between underemployment and job satisfaction was attenuated and rendered insignificant when participants reported higher levels of social support. Simple slope analyses of the interaction between underemployment and job satisfaction showed that the effect of social support tended to be more positive and different from zero at higher levels of underemployment. This indicates that for individuals with high social support, the relationship between underemployment and job satisfaction was not stronger (i.e., the slope was steeper) than for individuals with low social support.

## 4. Discussion

In the present study, we examined whether the perception of subjective underemployment influenced job satisfaction via work needs satisfaction in a sample of Turkish employees. We also tested whether social support plays a significant role as a moderator between underemployment and job satisfaction. The results showed that underemployment is negatively related to job satisfaction and that work needs satisfaction mediates the relationship between underemployment and job satisfaction (Hypothesis 1). In addition, social support moderates the relationship between subjective underemployment and job satisfaction such that the relationship is not significant when social support is higher (Hypothesis 2). In this section, we will begin by discussing the results obtained regarding the correlations between underemployment and other variables. Subsequently, we will interpret the outcomes of the model testing.

Based on our preliminary analysis, we identified a significant negative correlation between underemployment and job satisfaction, job needs satisfaction, and social support. However, it was noted that there was no significant relationship between the underemployment variable and job tenure. Primarily, we can assert that the negative relationship between underemployment and job satisfaction is an anticipated outcome. According to the Psychology of Working Theory, securing decent work significantly enhances individuals’ well-being and job satisfaction [19]. In this context, as outlined in the introduction, the contrast between the qualities associated with underemployment and the concept of decent work clarifies the observed negative correlation between underemployment and job satisfaction. Additionally, numerous studies have demonstrated a consistent negative association between employees’ perceptions of underemployment and their level of job satisfaction [20,21,36]. Consequently, it can be inferred that individuals’ perception of working in jobs that are lower than what they deserve exhibits a negative correlation with their overall job satisfaction.

Another significant outcome derived from our investigation into the interrelationships among variables was the recognition of a negative correlation between underemployment and the subdimensions of work needs. This finding corroborates the foundational tenets of the Psychology of Working Theory, which asserts that inadequate work situations hinder individuals from fulfilling their inherent work-related needs [19]. Furthermore, various studies have demonstrated that underemployment, including precarious work, exhibits negative associations with fundamental work needs, such as survival needs, autonomy, and relatedness [13,73]. Similarly, in a study conducted by Blustein and colleagues focusing on employment profiles, it was observed that profiles linked with underemployment exhibited lower satisfaction with competence, relatedness, and autonomy needs [74]. As a result, it is evident that the perception of underemployment, encompassing indecent work conditions, correlates negatively with employees’ satisfaction regarding their work-related needs. Thus, this finding suggests that the perception of underemployment hinders meeting work-related needs satisfaction.

Another finding from the research indicates a negative relationship between underemployment and social support. Several studies on underemployment have indicated a negative correlation between the perception of underemployment and levels of social support—a trend consistent with the findings of this study [1,75]. Social support refers to the relationships individuals establish within their social environment, such as family, friends, or significant others, and the support they receive from these connections [65]. While research on this topic remains limited, some scholars emphasize a negative correlation between underemployment and social relationships [21,22]. Thus, a plausible explanation for the negative association between underemployment and social support could be that individuals perceiving underemployment struggle to foster strong relationships within their environment, consequently hindering their effective utilization of available social support resources. 

In our study, we explored the correlation between underemployment and job tenure, finding no significant association between the two variables. While the existing literature offers limited insights into the relationship between job tenure and underemployment, the findings from various studies are inconsistent. While some studies suggest a negative relationship [76], the majority of research indicates no significant connection [77,78,79]. Consequently, further investigation is necessary to understand the dynamics between job tenure and underemployment fully.

As hypothesized, subjective underemployment is negatively associated with job satisfaction, as we elaborated on in the prediscussion of the relationship between these two variables. Moreover, the satisfaction of work needs serves as a mediator in the connection between underemployment and job satisfaction. This finding is consistent with the hypothesis of the PWT that the quality of work can affect work needs satisfaction and result in job satisfaction [19]. This result revealed that subjective underemployment affects job satisfaction negatively by lowering individuals’ ability to meet their work needs satisfaction. In other words, when individuals experience underemployment, they report that fewer of their work needs are met and thus experience less job satisfaction. We initially employed the five-factor structure of the work needs satisfaction scale to examine the significance of the mediation effect in the relationship between underemployment and job satisfaction. However, when this approach did not yield a significant result, we proceeded to reanalyze the data using the total score of the work needs satisfaction scale, similarly to previous studies [61]. Subsequently, we found that the mediation effect became significant when utilizing the total score of the WNSS. That the results turned out to be significant when we employed the total score instead of the subscales may be attributed to a number of factors. One of the possible reasons for this result is the high correlation among the work needs subscales, which may lead to a high degree of overlap among them. Furthermore, it is possible that fulfilling work-related needs holds greater significance for overall life satisfaction or well-being compared to one’s specific job experiences. 

Another possible explanation comes from the nature of job satisfaction. Various explanations exist regarding the nature of job satisfaction, with Kalleberg’s theory being one of the prominent frameworks [50]. Kalleberg suggests that job satisfaction is significantly influenced by the alignment between work values and job rewards, identifying six dimensions of values and rewards related to job satisfaction. These include intrinsic factors, such as the level of interest in the job and opportunities for skill development, as well as extrinsic factors like pay and work conditions. This suggests that job satisfaction is influenced by both intrinsic and extrinsic factors. However, Kalleberg states that more than one set of values and rewards may have common effects in predicting job satisfaction [50]. Similarly, work needs satisfaction comprises both internal and external dimensions, with survival needs representing external factors and elements like social contribution and autonomy representing internal needs [41]. Our study underscores the significance of multifaceted work needs satisfaction in predicting overall job satisfaction, aligning with Kalleberg’s notion of a complex structure. 

On the other hand, underemployment experience can play an inhibiting role in both the internal and external needs of employees. For example, Kim and Allan have emphasized that working in temporary jobs, which is one dimension of underemployment, can limit employees’ autonomy needs [13]. Similarly, such jobs can also negatively affect employees’ abilities to build relationships with their colleagues. Therefore, this situation demonstrates that underemployment can play a suppressive role in the internal factors of job satisfaction needs. Furthermore, it can be argued that the dimensions of underemployment related to low wages can also play a role in hindering employees’ external needs, such as survival. However, our findings revealed that although the subdimensions of underemployment and work needs satisfaction did not individually emerge as significant predictors of job satisfaction, their combined total scores did. This suggests that the aggregated influence of these dimensions, mediated by work needs satisfaction, significantly predicts overall job satisfaction. Thus, it underscores the importance of recognizing the mediating role of work needs satisfaction in facilitating the impact of underemployment on job satisfaction, rather than considering them solely as independent factors.

In summary, while the insignificance of individual subdimensions may seem confusing at first, the significant impact of total scores emphasizes the importance of considering the cumulative effects and interactions among various dimensions in understanding their influence on job satisfaction. This nuanced perspective provides valuable insights for both theoretical understanding and practical interventions aimed at improving job satisfaction in the context of underemployment.

Additionally, the results indicate that social support buffers the negative effect of perceived subjective underemployment on job satisfaction. When social support is high, there is no relationship between subjective underemployment and job satisfaction. This result is consistent with the PWT assumption that social support is a moderator variable that can buffer the negative effects of contextual factors [19]. In addition, this result can be explained by the cultural context. When we consider the collectivist characteristics of Turkish society [80,81], social support is more effective in people’s work lives [82]. Therefore, this finding is meaningful in terms of both PWT and cultural context.

## 5. Implications for Theory and Practice 

The current study contributes to the literature on the PWT and underemployment. While several studies have connected PWT and underemployment [13,83], to the best of our knowledge, no research has been conducted on the outcomes of subjective underemployment in the PWT framework. Our findings are significant when considering the PWT’s emphasis on quality of work because testing different types of employment in the PWT framework may help us understand how well workers are able to meet their work needs [19,84]. In addition, our study’s findings are important because, unlike other studies that tested individual factors (such as meaningful work) in buffering underemployment [12], we revealed the moderating effect of social support as a contextual factor that can buffer the negative impact of subjective underemployment. Therefore, it is crucial to focus on social support resources for underemployed workers, especially those who cannot fulfill their work needs and who experience job dissatisfaction due to underemployment. In addition, we examined subjective underemployment using a sample of Turkish employees. This is important because cultural differences can be critical in career counseling [85]. For this reason, career counselors could encourage clients experiencing underemployment to use social support resources, especially when working with clients with a collectivist cultural background. Career counselors can thus be more effective in helping clients cope with the adverse effects of underemployment. Finally, the study indicated that underemployment as an employment status can fit the PWT model, and counselors can consider it within the PWT framework. In other words, counselors can encourage clients to explore their awareness of how their employment status plays a role in their work needs satisfaction and job satisfaction. Moreover, counselors can assume a critical role in assisting clients to mobilize sources of social support outside of work, thereby mitigating the adverse effects of underemployment.

## 6. Limitations and Future Directions

The present study has several limitations. Firstly, due to the utilization of a cross-sectional design and reliance on self-report instruments, causal inferences cannot be made, and our findings may have been influenced by participants’ current perceptions. We thus recommend investigating subjective underemployment based on the PWT framework in longitudinal studies. Second, it is worth noting that there were variations in educational attainment among participants across different occupational groups. By way of illustration, cashiers exhibit a level of educational attainment below the mean, while academic personnel demonstrate a notably higher level of educational attainment in comparison to the average. Given the uneven distribution among occupations, we refrained from incorporating professional disparities or participant education levels into the model. Nevertheless, it is imperative to acknowledge the potential influence of educational attainment and occupational distinctions on the relationships between variables. In future research endeavors addressing underemployment, careful consideration of both educational levels and occupational disparities is advised for a more comprehensive understanding of the phenomenon. Third, our findings verified the moderating effect of social support between subjective underemployment and job satisfaction. It should be noted, however, that the data were collected from Turkish employees, meaning that this result may be attributable to cultural context. Therefore, the moderating effect of social support on subjective underemployment should be tested in cultures with more individualistic characteristics.

Finally, we propose the integration of subjective underemployment into the Psychology of Working Theory. Hence, future studies may identify new models that include underemployment within the framework of PWT.

## Figures and Tables

**Table 1:** Descriptive statistics and correlations of study variables.

Variables	1	2	3	4	5	6	7	8	9	10	Mean	*SD*
1. SU	-										115.41	50.31
2. JS	−0.25 **	-									22.98	5.01
3. WNS	−0.41 **	0.50 **	-								105.66	22.93
4. WNS—Survival	−0.41 **	0.32 **	0.72 **	-							22.12	6.10
5. WNS—Social contribution	−0.34 **	0.35 **	0.79 **	0.61 **	-						18.44	4.01
6. WNS—Competence	−0.36 **	0.44 **	0.85 **	0.50 **	0.61 **	-					22.66	5.66
7. WNS—Relatedness	−0.31 **	0.49 **	0.86 **	0.47 **	0.59 **	0.72 **	-				21.80	6.22
8. WNS—Autonomy	−0.28 **	0.39 **	0.85 **	0.48 **	0.60 **	0.63 **	0.69 **	-			20.64	6.43
9. SS	−0.14 *	0.23 **	0.43 **	0.32 **	0.29 **	0.40 **	0.36 **	0.38 **	-		66.32	14.75
10. Job tenure	−0.01	0.00	0.02	0.03	0.08	−0.05	−0.01	0.06	−0.14	-	13.70	8.08
Skewness	0.38	−0.58	−0.67	−0.92	−0.90	−1.09	−0.14	−0.70	−0.70	1.07		
Kurtosis	−0.66	−0.18	−0.22	0.13	0.39	0.86	0.47	−0.30	−0.19	1.53		

* *p* < 0.05, ** *p* < 0.001; SU: subjective underemployment, JS: job satisfaction, WNS: work needs satisfaction, SS: social support, *SD*: standard deviation.

**Table 2:** Mediation and moderation analyses.

**Dependent Variable: Job Satisfaction**							
				**Confidence Interval**			
	**B**	**SE**	***t***	**LL 95% CI**	**UL 95% CI**	**R^2^**	**F**
Constant	14.784 **	1.601	9.236	11.638	17.930	0.26	31.460 **
SU	−0.015 *	0.007	−2.328	−0.028	−0.002		
WNS	0.103 **	0.010	9.988	0.083	0.124		
SS	−2.052	1.073	−1.913	−4.160	−0.056		
SU × SS	0.018 *	0.008	2.188	0.002	0.035		
**Covariate Variable: Job Tenure and Gender**							
Job tenure	0.005	0.026	0.201	−0.046	0.057		
Gender	−0.609	0.430	−1.416	−1.454	0.236		
**Moderator Effect of Social Support**							
	**B**	**SE**	***t***	**LL 95% CI**	**UL 95% CI**		
Low	−0.015 *	0.007	−2.328	−0.028	−0.002		
High	0.003	0.006	0.505	−0.008	0.014		
Mediation Effect between SUS and JSS of WNSS ***							
	**Indirect Effect**	**SE**		**LL 95% CI**	**UL 95% CI**		
WNS	−0.019 **	0.029		−0.026	−0.014		

* *p* < 0.05, ** *p* < 0.001, *** Number of bootstrap samples for percentile bootstrap confidence intervals: 5000; SS: social support, WNS: work needs satisfaction, SU: subjective underemployment, JS: job satisfaction, SE: standard error.

## Data Availability

The datasets generated and analyzed during the current study are available from the corresponding author on reasonable request.
